# The Human Microbiome and Its Impacts on Health

**DOI:** 10.1155/2020/8045646

**Published:** 2020-06-12

**Authors:** Grace A. Ogunrinola, John O. Oyewale, Oyewumi O. Oshamika, Grace I. Olasehinde

**Affiliations:** Department of Biological Sciences, Covenant University, Ota, Ogun State, Nigeria

## Abstract

The human microbiome comprises bacteria, archaea, viruses, and eukaryotes which reside within and outside our bodies. These organisms impact human physiology, both in health and in disease, contributing to the enhancement or impairment of metabolic and immune functions. Micro-organisms colonise various sites on and in the human body, where they adapt to specific features of each niche. Facultative anaerobes are more dominant in the gastrointestinal tract, whereas strict aerobes inhabit the respiratory tract, nasal cavity, and skin surface. The indigenous organisms in the human body are well adapted to the immune system, due to the biological interaction of the organisms with the immune system over time. An alteration in the intestinal microbial community plays a major role in human health and disease pathogenesis. These alterations result from lifestyle and the presence of an underlying disease. Dysbiosis increases host susceptibility to infection, and the nature of which depends on the anatomical site involved. The unique diversity of the human microbiota accounts for the specific metabolic activities and functions of these micro-organisms within each body site. It is therefore important to understand the microbial composition and activities of the human microbiome as they contribute to health and disease.

## 1. Introduction

The human microbiota is defined as a set of organisms inhabiting and interacting with the human body [1]. The various interactions may be commensalistic, mutualistic, or pathogenic. The human microbiome is referred to as the genomic content of organisms (microbiota) inhabiting a particular site in the human body. micro-organisms colonise various anatomical body sites such as the skin, the mucosa, gastrointestinal tract, respiratory tract, urogenital tract, and the mammary gland. They form a complex and discrete ecosystem that adapts to the environmental conditions of each niche [2]. From childbirth, a steady interaction (symbiosis) between the human body and its indigenous microbiota begins. These interactions play important roles in maintaining general health and wellbeing. Through coevolution, organisms make up the microbiota, they actively adjust to their specific habitats and reside in their respective niches within the human body [3–5]. As a result of their biological activities, these organisms are identified as part of the body, leading to various changes from conception until death. The human microbiome is constantly evolving in response to host factors. Factors such as age, nutrition, lifestyle, hormonal changes, inherited genes, and underlying disease are major determinants of the human microbiome at any given point in time. However, an alteration in the makeup of the human (dysbiosis) microbiota can lead to life-threatening illnesses [2]. A balanced microbiota has shown to play an important role in health sustenance [2]. The largest concentration of the human microbiome is found in the gut. These organisms are the major players in maintaining and sustaining the health of humans. Past studies on the human microbiome project have illustrated that changes in the immune environment may be directly linked to a dysbiotic flora of the gut. Also, life-threatening health conditions ranging from cancer, cardiovascular disease, bowel inflammatory disease and difficult-to-treat bacterial infections due to antibiotic resistance have also been linked with dysbiosis. [6, 7].

In general, this work aims to review and discuss the impact of the human microbiome on human disease and on maintaining health.

## 2. The Human Microbiome and Disease

### 2.1. Cancer

**Figure 1:**
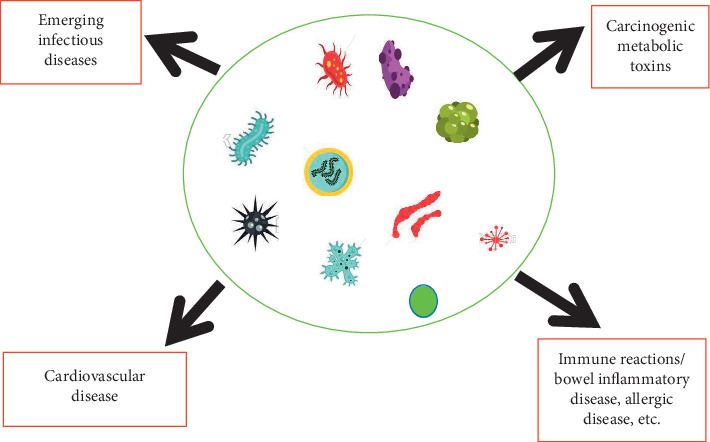
Dysbiotic flora and its impact on human health. Carcinogenic metabolic toxins produced from dysbiotic flora may trigger the progression of cancer and immune reaction in the gastrointestinal tract. In addition, hepatic oxidation of trimethylamine to trimethylamine N-oxide contributes to cardiovascular and emerging diseases.

**Figure 2:**
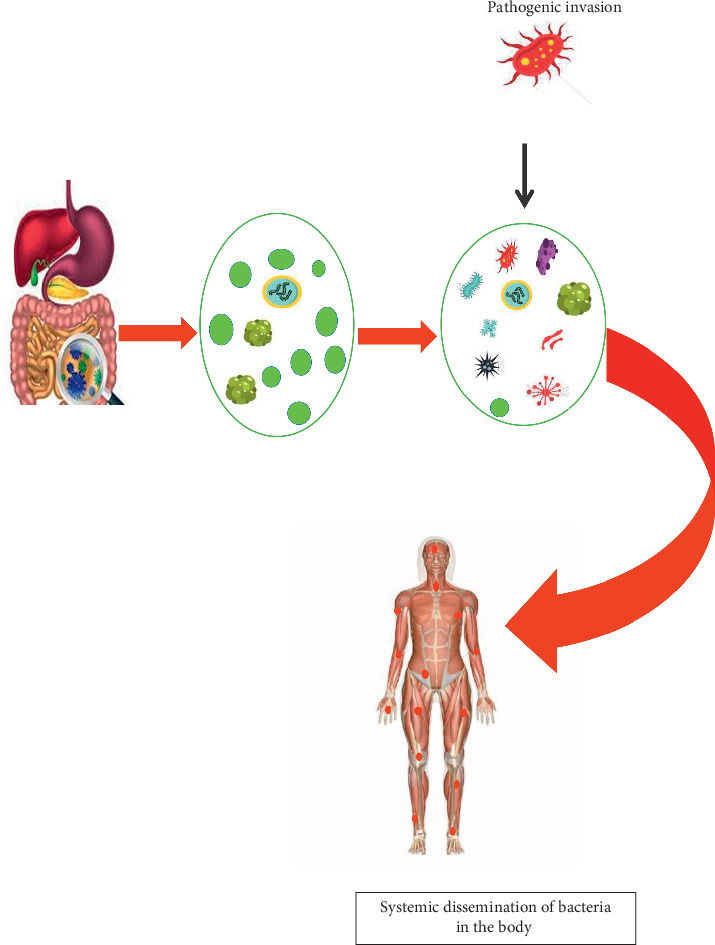
An alteration in the gut microbiota leads to systemic translocation of organisms from damaged gut epithelium to other extraintestinal sites.

The gut microbiota plays a significant role in affecting the well being of its host [8]. Studies on the interplay of microbial communities and their respective host suggest that these organisms carry out biochemical activities influencing carcinogenesis, tumour development, and response to immune therapy as shown in Figure 1 [8]. According to a well-studied model on factors that may contribute to dysbiosis in the gut, continuous intra-abdominal infections, antimicrobial drugs, or both may lead to an increased risk of colorectal cancer. Also, end products released by the gut microbiota affect the intestinal cell coverage, encouraging carcinogenesis or suppressing tumourigenesis [9]. Aside from colorectal cancer, the microbiota of the intestinal tract has shown to play a role in extraintestinal cancer such as hepatocellular carcinoma through systematic dissemination of these organisms to other parts of the body (Figure 2) [2]. In addition, *H. pylori* contributes to the risk of gastric cancer in humans. Recent findings on the interplay of the human microbiome and cancer point out *Fusobacterium* and *Clostridium* being overrepresented in individuals having gastric cancer [10]. In the case of breast cancer, environmental and host factors directly influence the progression of cancer in the breast. However, bacterial communities could also induce breast cancer. Individuals with breast cancer have been more likely to have *Bacillus,* members of the Enterobacteriaceae, and *Staphylococcus in* their breast tissue as compared to healthy individuals. Furthermore, *Escherichia coli* and *Staphylococcus epidermidis* isolated from patients having cancer triggered a double-stranded break in the DNA of HeLa cells. *Lactobacillus* spp. which contributes to diverse health benefits was not found in the breast tissue of individuals with breast cancer [11]. Prostate cancer has been implicated with a higher population of *Bacteroides massiliensis*. An alteration in the human microbiota has contributed to the complex interactions between cancer and the human microbiota [10].

### 2.2. Inflammatory Bowel Disease (IBD)

The accumulation of disease-causing organisms induces or triggers an abnormal immune response against body tissues. This, in fact, contributes to autoimmune diseases, bowel inflammatory disease, and other life-threatening conditions [12]. Due to the coevolution of the human microbiota and the immune system, a balanced and systematic interaction occurs over time. However, an alteration in the host-microbiota affects this interaction leading to impairment in immune response which may result in inflammatory disorder [13]. Sunil et al. [14] describe the relationship of the gut microbiome with a compromise in the integrity of the gastrointestinal barrier in inflammatory bowel disease (IBD). Barrier around epithelial cells forms a tight junction, which separates tissue space and controls the movement of solutes across the epithelium. The barrier function of the intestine may be affected by a damaged mucus layer, leading to a defective cell linkage attachment [15]. A reduction in gut Firmicutes leads to increased levels of proinflammatory cytokines (IL12, IFN-*γ*) and reduced anti-inflammatory cytokine (IL-10) levels [14]. Certain helminth infections were associated with anti-inflammatory organisms that prevent inflammatory bowel disease in IBD susceptible mice [10].

### 2.3. Cardiovascular Diseases

Production of metabolites by the gut microbiota does not only affect the gut but also act systemically. The production of trimethylamine N-oxide (TMAO) metabolites by certain gastrointestinal organisms may be implicated in heart disease [16]. The gut microbiota actively metabolizes trimethylamine from diets rich in l-carnitine, choline, and phosphatidylcholine to trimethylamine N-oxide (TMAO) by hepatic flavins containing monooxygenase. TMAO affects lipid transportation in the body and also induces the release of precursors which promote foam cell formation and hardening of the arteries in animal models [16]. Intestinal dysbiosis has been discovered to be associated with cardiovascular diseases. Kho and Lal performed a clinical study on two categories of individuals: individuals with low risk of cardiovascular disease and those with a risk of cardiovascular disease. In their findings, a disrupted intestinal flora was associated with individuals having a higher risk of cardiovascular disease [17, 18]. Overrepresentation of certain organisms has been found to contribute to cardiovascular diseases. Faecal transplants from hypertensive patients that had overexpressed *Prevotella* and *Klebsiella* increased blood pressure in germ-free mice animal models. Furthermore, the faecal microbiota of hypertensive mice showed a significant increase of the Firmicutes to Bacteriodetes ratio in their stool [18, 19].

### 2.4. Systematic Infections Resulting from Bacterial Translocation

Systematic infection occurs by the continuous movement of bacteria in human hosts from the intestinal mucosa to other extraintestinal sites as shown in Figure 2. The risk of a systemic disease from translocating organisms is higher in immune-compromised individuals who are hospitalized, undergoing surgery, or in trauma [19]. Damage in the epithelium of the gut and the abuse of antibiotics disrupts the microbiota, leading to an increase in facultative anaerobes and a defect in the host immune responses. Some examples of translocating organisms associated with systemic infections include *E. coli, K. pneumoniae, Enterobacter* spp.*, P. mirabilis, Enterococcus* spp.*, Streptococcus* spp., and *C. albicans* [19]. In addition, microbial translocation contributes to systemic infection by the production of uremic toxins. This discovery suggests that an alteration in the microbiota of the gut may lead to the synthesis of nitrogenous compounds which affect the integrity of the epithelial tight junction, allowing the transfer of these organisms and its toxins to other parts of the body. Uremic toxins produced by dysbiotic flora activate systemic inflammatory responses which is a trigger to several diseases [15]. In 2015, a clinical study discovered a link between gut microbiota and chronic kidney disease. They observed the presence of translocation gut microbiota in individuals on hemodialysis [16].

## 3. The Human Microbiome and Allergic Diseases

Possible mechanism of the human microbiome in association with allergic diseases has been identified [12]. Although, little is known on the effects of the lungs microbiota on immune regulation of the respiratory tract [20]. However, the respiratory tract is greatly shaped by a balanced gut microbiome that affects the mucosa of the lungs. A dysbiotic flora directly affects the microbiome of the lungs through microaspiration, and this increases the occurrence of respiratory diseases in humans. Renz et al. illustrated this finding in germ-free mice. Experimental mice were devoid of an immune regulatory network which induced respiratory and allergic diseases [21].

Caesarean (CS) delivery of neonates has also been identified as a risk factor for allergic diseases. The absence of normal maternal flora during CS predisposes children to such diseases [22]. Molecular-based studies have revealed that CS-delivered children have lower counts of healthy flora (Bacteriodetes) in their gut [6]. This reduces the anti-inflammatory activities of Bacteriodetes and contributes to local tissue inflammation (asthma and allergic rhinitis) triggered by genetic and environmental factors [12]. A recent epidemiological study reported a significant association between dysbiotic gut flora and the production of allergic antigen (IgE) resulting in airway disease in children [23]. Additional studies establish that children with lower microbial diversity of *Bifidobacterium*, *Akkermansia*, and *Faecalibacterium* were susceptible to multiple allergen respiratory sensitivity (polysensitisation) and may contribute to asthma at the age of 4 [24]. A review by Huang et al. further supports these findings. Germ-free mice were more susceptible to allergic airway disease. After microbial colonization, the susceptibility was reversed and a reduced allergen sensitivity was observed. Clinical studies on the incidence of allergies in Europe showed that farming environments containing diverse microbial communities had a lower rate of airway allergies [25]. The mechanism behind this phenomenon has been linked to the activation of the innate immune system in the epithelial cell of the respiratory tract. Exposure to farm dust containing microbial diversities of *Acinetobacter lwoffii* F78 and *Lactococcus lactis* G121 has proven to reduce respiratory inflammation in mice [25, 27].

## 4. The Human Microbiome in Health Sustenance

### 4.1. Maintenance of Homeostasis

**Figure 3:**
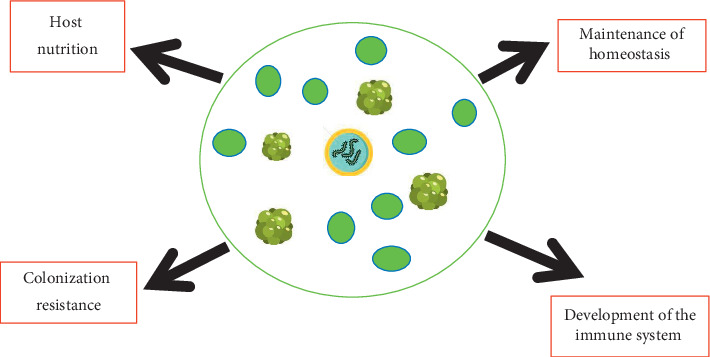
Symbiotic interaction between eubiotic flora and the human body results in the maintenance of homeostasis, regulation, and development of the immune system, hosts nutrition, and colonization resistance.

The human microbiome plays important roles in the maintenance and development of the human body (Figure 3). These organisms are responsible for launching the immune system, affecting inflammatory homeostasis and immune regulation in neonates and young children [14]. In 2015, Melli in his study reviewed that children who develop allergies later in life were identified to have a higher prevalence of Bacteroidaceae and anaerobic bacteria with a lower count of *Bifidobacterium adolescentis*, *Bifidobacterium bifidum*, and *Lactobacillus* spp. [26, 27]. Continuous research studies on microbiome have also identified that these organisms interact with and degrade external contaminants such as heavy metals, polycyclic aromatic hydrocarbons, pesticides, ochratoxins, plastic monomers, and organic compounds [28]. Following renal filtration in the kidney, the toxins removed from the bloodstream are stored within the bladder which provides substrates and a conducive environment for the urinary tract microbiota to deactivate toxic substances [29]. The activities of these organisms interplay in the outcome of an infection. In the female genital tract, the protective mechanism is initiated by indigenous microbial flora which is responsible for inducing innate immunity, including the secretions containing cytokines, antimicrobial peptides, and inhibitory substances [29].

### 4.2. Development of Host Immune System

Through coevolution of indigenous microbiota and the immune system, immune responses are developed and enhanced by the immune system's ability to differentiate between harmful pathogens and commensal organisms that must be maintained [30]. In the gut, the composition of the microbiota influences the developmental aspects of the adaptive immune system; therefore, the mammalian immune system, which is responsible for controlling micro-organisms, is shaped by the human microbiota [13]. In recent studies on the functions of the human microbiome, it has been illustrated that the absence of these organisms or early alteration of commensal organisms may result in exacerbated type II immunity and allergies as a result of an abnormal immune functionality. In children, for example, alterations to the microbiota through epigenetic influences such as caesarean births, an increasingly sedentary lifestyle, environmental pollution, and Western-type diets have been linked with an increase in cases of childhood allergic rhinitis [7, 22]. Probiotics, breastfeeding, lifestyle changes such as allowing children to play out in the morning sunshine (to promote vitamin D production), and allergen-specific immunotherapy have been proposed as factors promoting the development of the immune system of and the prevention of atopy in children [22]. The gut microbiota is responsible for activating the proinflammatory Th17cells and regulatory T-cells (Tregs) in the intestine [10]. In addition, the human microbiota has significant influence on innate immunity. An example is neutrophil ageing which reduces proinflammatory properties in the body [30]. These organisms stimulate neutrophil ageing through Toll-like receptor- (TLR-) and MyD88-mediated signalling pathways. Microbial alteration leads to reduced circulation of aged neutrophils and in return results in inflammation-related organ damage in models of sickle cell disease or endotoxin-induced septic shock. Therefore, these organisms actively control disease-promoting neutrophil which is necessary for inflammatory diseases [30]. Furthermore, the intestinal microbiota helps in the defence against pathogenic organisms. They promote colonization resistance and the synthesis of antimicrobial compounds against invading pathogens. For example, a balanced gut microbiome may be responsible for regulating antibodies (CD_8_-T cells and CD_4_ cells) which respond to the invasion of influenza virus in the respiratory tract [12]. Also, the intestinal microbiota helps to improve and maintain the gastrointestinal functions [31]. The high concentration of organisms in the gut remains a problem to the intestinal immune system, as the immune system needs to accept commensal microbiota and dietary antigens while also retaining its ability to eradicate pathogens. The activation of colonic regulatory T-cells (Tregs) is important in developing immune homeostasis [14]. There are two types of Tregs: the thymus-derived and peripherally derived Tregs (pTregs). There is complexity in the differentiation of these two immune responses, but they have an essential role in immune regulation [14]. However, the pTregs, in particular, require microbiota to be active in the colon.

### 4.3. Host Nutrition

The colonic microbiota makes significant contributions to the nutritional requirements of their host [32]. These organisms actively break down complex dietary constituents that are indigestible (complex carbohydrates) by the intestinal cells making complex food materials readily available for absorption and assimilation. In the digestive tract, major end products of carbohydrates and amino acids are the short-chain fatty acids (SCFAs) which include acetic, propionic, and butyric acids [33]. These three dietary constituents, upon absorption by colonic mucosa, serve as energy sources and precursors for the synthesis of mucosal lipids and stimulate cell growth of the epithelial cell resulting in maintenance of gut integrity [19]. Colonic microbiota protects the large bowel against cancer by the production of butyrate from the fermentation of complex dietary constituents [9]. These important microbial activities in the colon have resulted in the provision of essential nutrients which are not readily accessible but are required for colonic health sustenance [33]. In Africa, mothers and infants have been discovered to contain a high level of Bacteriodetes and SCFAs in their stool as compared with the European infants whose mothers consume Western diets low in SCFAs. Studies have shown that the consumption of traditional and fermentable carbohydrate may have contributed to the prevalence of healthy gut microbiome [12]. Another important function of the colonic microbiota is the provision of vitamins necessary for host development. Intestinal bacteria such as *Bifidobacterium* spp., *Bacteroides* spp., and enterobacteria are responsible for the production of vitamins [32]. Vitamin K, for instance, is an important coenzyme responsible for the synthesis of several clotting factors which include prothrombin (a deficiency of this leads to delayed blood clotting and excessive bleeding). Also, folic acid is an important precursor for DNA and RNA synthesis. Finally, they are involved in the synthesis of red and white blood cells [19]. Today, probiotics containing *lactobacillus* or *Bifidobacterium* are used in treatments of allergic diseases. Findings from the use of probiotics as treatment options have revealed that an enhanced immunomodulatory effect is achieved by reducing or inhibiting antigen-inducing T-cell activation and also suppressing cell signalling protein (tumour necrosis factor (TNF) involved in systemic inflammation [12].

## 5. Conclusion and Recommendation

The study of the human microbiome is important, and it gives an in-depth understanding of the interplay between humans and its indigenous microbiota. This gives valuable insight into further research studies in optimizing these organisms to combating life-threatening diseases. It is important to note that the continuous use of broad-spectrum antibiotics may disrupt the human microbiota. This results in an imbalance of the indigenous microbial community paving way for invading pathogens. However, treatments with the use of pre and probiotics should be encouraged. Hence, more research should be focused on the use of probiotic therapy in the treatment of infectious disease. In addition, further studies should emphasize on the effects of the human microbiome on mental health and also the impacts of mycobiome and the virome community on indigenous microbiota as they may contribute to dysbiosis.

## Data Availability

No data were used to support this study.
